# A Very‐High Frequency Solution‐Processed Organic Field‐Effect Transistor With Improved Voltage‐Normalized Frequency of Transition

**DOI:** 10.1002/advs.77513

**Published:** 2026-09-13

**Authors:** Tommaso Losi, Kyle van Oosterhout, Martino Cambiaggio, Martin Heeney, Qiao He, Hans Kleemann, Alessandro Luzio, Eugenio Cantatore, Mario Caironi

**Affiliations:** ^1^ Center for Nano Science and Technology Istituto Italiano di Tecnologia Milano Italy; ^2^ Department of Electrical Engineering Eindhoven University of Technology Eindhoven The Netherlands; ^3^ Department of Physics Politecnico di Milano Milano Italy; ^4^ Division of Physical Sciences & Engineering Chemistry Program King Abdullah University of Science and Technology (KAUST) Thuwal Saudi Arabia; ^5^ College of Education Sciences The Hong Kong University of Science and Technology (Guangzhou) Guangzhou China; ^6^ Dresden Integrated Center for Applied Physics and Photonic Materials (IAPP) Technische Universität Dresden Dresden Germany

**Keywords:** direct‐writing, frequency of transition, organic blend semiconductor, organic electronics, very‐high frequency transistors

## Abstract

Achieving very‐high (VHF) and ultra‐high frequency (UHF) operation in organic field‐effect transistors (OFETs) would expand their field of application to wireless communication. Such vision remains a major challenge, with OFETs mostly limited to High‐Frequency bandwidth (3 – 30 MHz) due to intrinsic limitations, such as contact resistance and charge mobility, and fabrication‐related parasitism, especially when adopting solution‐based approaches. Here, we report on p‐type OFETs exhibiting a frequency of transition (*f_T_*) up to 135 MHz at *V_gs_* = *V_ds_* = −22 V, improving the voltage normalized *f_T_* (*f_T_*/*V^2^*) for VHF organic transistors with *f_T_* > 100 MHz to 0.28 MHz V^−2^. The devices were realized through a combination of direct‐writing and solution‐processing techniques employing an air‐stable high‐mobility semiconductor blend based on 2,7‐dioctyl[1] benzothieno[3,2‐*b*][1]benzothiophene (C_8_‐BTBT) and poly(indaceno‐dithiophene‐*co*‐benzothiadiazole) (C_16_IDT‐BT). Femtosecond‐laser sintering enabled submicron gate overlaps (∼ 0.4 µm), minimizing width‐normalized parasitic gate capacitances (*C_g_*/W ≈ 2.5 pF cm^−1^). DC and AC device characteristics can be approximated using compact models, and theoretical simulations indicate that these OFETs could be potentially used to fabricate rectifiers with a ‐3 dB cut‐off frequency of 200 MHz. These results represent a significant step toward high‐speed organic electronics, establishing a pathway for wireless systems fabricated through scalable solution‐based processes.

## Graphical abstract

Increasing the frequency at which organic electronics operate while limiting the operation voltage is key to enabling future sustainable wireless applications. This work shows p‐type organic field‐effect transistors based on a molecularly doped, small molecule/polymer blend (C_8_‐BTBT:C_16_IDT‐BT), fabricated via direct‐writing and solution‐processing, achieving a transition frequency of 135 MHz of at −22 V. This result demonstrates the potential of organic semiconductors and solution‐based approaches for wireless distributed electronics.

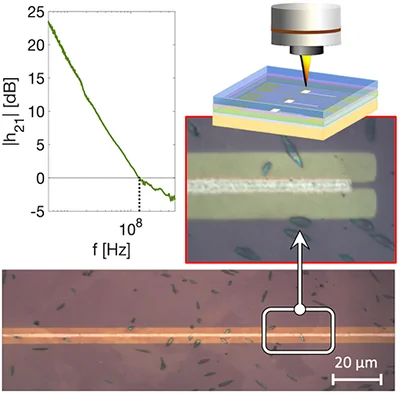

## 1 Introduction

Organic microelectronics has undergone numerous advances, unlocking new possibilities in different application fields such as health‐care, bio‐sensing, agritech and space [1, 2, 3, 4, 5, 6]. It provides compatibility with large‐area, energy‐efficient, low‐cost, solution‐based fabrications, as well as with lightweight and flexible substrates, highly desirable for sustainable wearable technologies [7, 8, 9, 10]. However, some key functionalities are still lacking, severely limiting potential applications. One of these is the capability to enable radio frequency wireless communication, a fundamental feature to collect and transmit data, as required in many communication protocols [11, 12]. In particular, achieving ultra‐high frequency operation UHF (300 MHz—3 GHz) is necessary for the development of sustainable Internet of Things (IoTs) devices, and e.g., for potential exploitation in satellite communications in Low Earth Orbit. Currently, the operational bandwidth of organic electronics is mostly limited to the High‐Frequency range (3—30 MHz) [11], owing to the limit imposed by the frequency of transition (*f_T_*) of the main building block of organic microelectronic circuits, i.e., the organic field‐effect transistor (OFET). *f_T_* is the figure‐of‐merit mostly used to assess the AC performance of FETs [11], expressed as follows:

(1) $$f_{T}=\frac{g_{m}}{2\pi \left(C_{g\left(ch\right)}+C_{g\left(ov\right)}\right)}=\frac{\mu_{eff}\left|V_{gs}-V_{T}\right|}{2\pi L_{C}\left(\alpha L_{C}+2L_{ov}\right)}\;$$

where *g_m_* is the transistor transconductance, *C_g(ch)_* and *C_g(ov)_* the capacitances related to the transistor channel and geometrical overlap parasitism between gate and source/drain electrodes, respectively, *µ_eff_* the effective charge carrier mobility [13], *V_T_* the threshold voltage, *L_C_* and *L_ov_* the channel and overlap lengths, respectively, and α is a parameter ranging from 2/3 to 1 according to the mobility dependence on the bias voltage [11, 14, 15]. The three main obstacles in enabling UHF operation in OFETs are: (i) charge injection inefficiencies, i.e., a too high contact resistance (*R_C_*), which limits *µ_eff_* in downscaled OFETs [11, 16, 17, 18, 19], (ii) the limited resolution of low‐cost solution‐based patterning methods, which limit the *L_C_* scaling and result in larger overlaps [11, 20, 21, 22], (iii) the low thermal conductivity of organic materials, leading to severe self‐heating effects [11, 23, 24, 25, 26]. To date, the highest ever reported value of *f_T_* for organic transistors is 160 MHz [27], falling within the Very‐High Frequency (VHF, 30—300 MHz) range, relevant for radio communications, navigation systems and telemetry [27, 28, 29, 30]. This result was achieved by A. Perinot et al. with an n‐type OFET based on the polymer poly[N,N’‐ bis(2‐octyldodecyl)‐naphthalene‐1,4,5,8‐bis(dicarboximide)‐2,6‐diyl]‐alt‐5,5’‐(2,2’‐bithiophene) (P(NDI2OD‐T2)), operating at 40 V. It is the only case of an organic transistor with *f_T_* exceeding 100 MHz, and it was obtained exclusively through solution‐based direct‐written fabrications. However, the poor ambient stability of P(NDI2OD‐T2) [31, 32], as well as the high bias voltages employed, place this result far from real application scenarios. In fact, the unprecedented *f_T_* value was obtained adopting relatively low areal capacitances, which facilitates the reduction of gate capacitive parasitism. However, since *g_m_* is inversely proportional to the channel capacitance, such a choice forces a proportional increase of the gate voltage to maintain an equivalently high transconductance. Considering that *f_T_* is proportional to the applied bias, a voltage‐normalized transition frequency (*f_T_/V* or *f_T_*/*V^2^*) is a more balanced figure‐of‐merit to evaluate the relative performance of different OFET technologies [11, 33, 34]. As a comparison, the group of H. Klauk demonstrated 21 MHz operation at only ‐3 V with a p‐type transistor based on the vacuum evaporated small‐molecule 2,9‐diphenyl‐dinaphtho[2,3‐*b*:2′,3′‐*f*] thieno[3,2‐*b*]thiophene (DPh‐DNTT), reaching a *f_T_/V^2^* of 2.3 MHz V^−2^ [35], which exceeds the 0.1 MHz V^−2^ of the VHF solution‐processed device. Low‐voltage operation was achieved through the implementation of an ultra‐thin, yet robust gate dielectric with high areal capacitance. Nevertheless, achieving high absolute *f_T_* values, beyond the HF band, remains challenging for devices with large dielectric capacitance, as self‐heating effects and bias‐induced instabilities can significantly degrade performance.

In this work, we show a combination of direct‐writing techniques and solution‐based processes to realize VHF OFETs based on a solution‐processed semiconductor with a substantially improved voltage normalized *f_T_*. The realized OFETs are based on one of the best performing solution‐processable organic semiconductors, the blend of the small molecule C_8_‐BTBT (2,7‐dioctyl[1] benzothieno[3,2‐*b*][1]benzothiophene) [36] and the co‐polymer C_16_IDT‐BT (poly(indacenodithiophene‐*co*‐benzothiadiazole)) [37]. We successfully adopted such a high‐performing blend in engineered 1.1 µm short‐channel devices, retaining an effective charge mobility of 0.6 cm^2^ V^−1^ s^−1^. The OFETs are endowed with a dielectric featuring both a relatively high areal capacitance, to enable ∼ 20 V of operation, and robustness to fs‐laser sintering of micron‐scale gates. As a result, overlap lengths are scaled down to ∼ 0.4 µm, obtaining a drastic reduction in capacitive parasitism. A total width‐normalized gate capacitance of only 2.5 pF cm^−1^ pushes *f_T_* within the 100 MHz range at applied voltages in the range of 20 V. With our approach, the highest *f_T_* obtained is 135 MHz at *V_gs_* = *V_ds_* = ‐22 V, scoring the best *f_T_*/*V^2^* of 0.28 MHz V^−2^ for VHF OFETs with *f_T_* > 100 MHz and the best *f_T_* value for p‐type OFETs in general. This result marks a relevant step toward UHF organic microelectronics at viable operation voltages, obtained with scalable processes.

## 2 Results and Discussion

**FIGURE 1:**
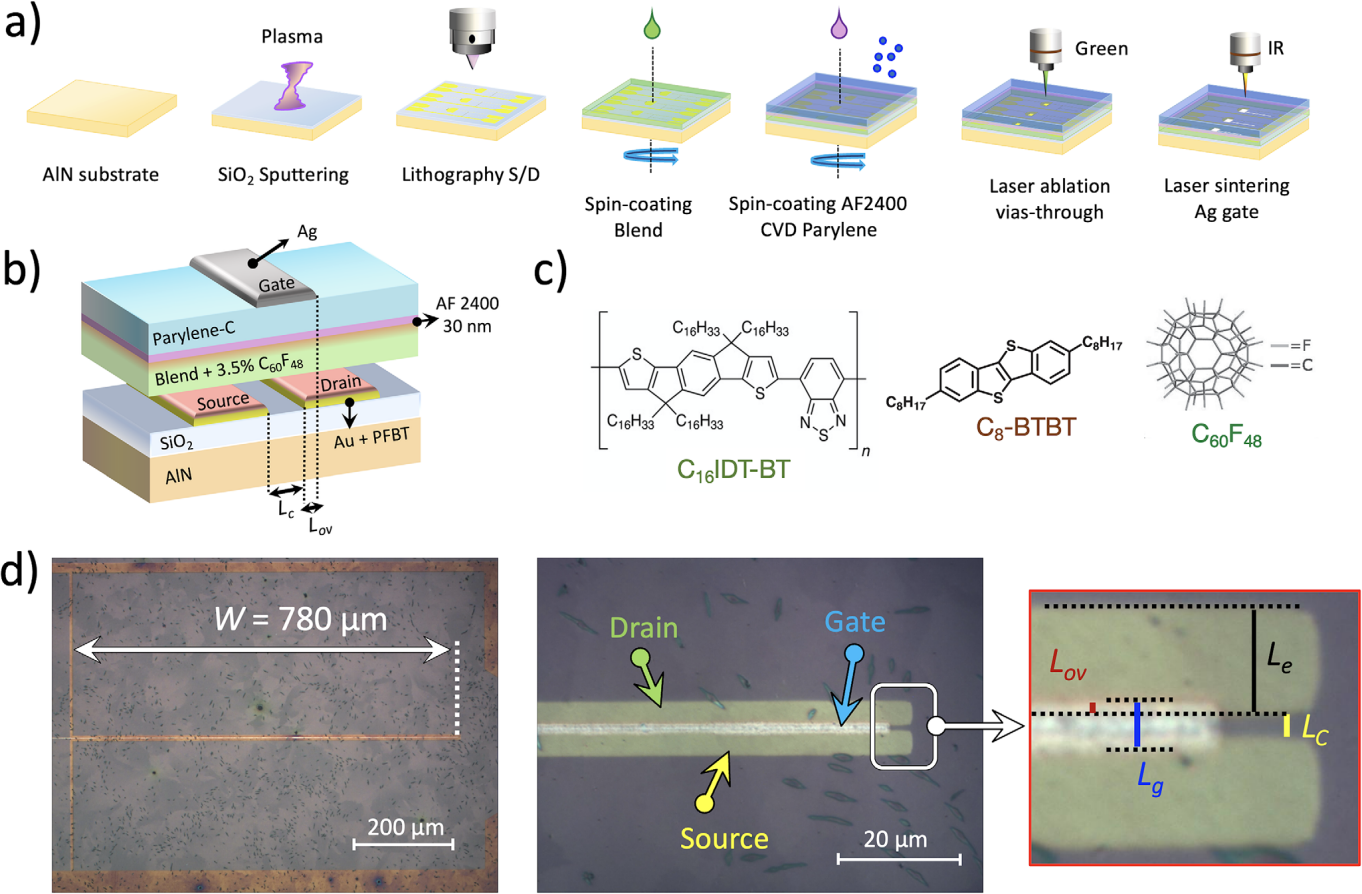
(a) Scheme of the process flow used for VHF OFETs fabrication. (b) Sketch of the transistor architecture. (c) Chemical structures of C_16_IDT‐BT, C_8_‐BTBT and C_60_F_48_. (d) Optical polarized images of transistors based on doped C_16_IDTBT:C_8_‐BTBT with laser‐sintered gate electrodes. In the image on the right, *L_C_* is the channel length, *L_e_* is the source/drain electrode length, *L_g_* is the gate length, and *L_ov_* is the overlap length.

We fabricated the VHF OFETs in a top‐gate bottom‐contacts configuration, as illustrated in Figure 1a,b. We used aluminum nitride substrates (AlN) to alleviate self‐heating phenomena, which are known to cause important thermal damages in short‐channel OFETs based on C_8_‐BTBT:C_16_IDT‐BT blend [26, 27]. The AlN substrates employed in this work are characterized by an average thermal conductivity of 165 W m^−1^ K^−1^, allowing efficient thermal dissipation during device operation [27]. On top of the substrates, a 300 nm thick interlayer of SiO_2_ was deposited by sputtering to protect AlN toward subsequent fabrications and to offer a smoother interface for semiconductor deposition. The bias stability of OFETs fabricated on AlN/SO_2_ substrates was verified on reference short‐channel devices in full overlap (Figure S1). Source and drain gold contacts were patterned by direct‐written maskless photolithography to obtain a channel length of 1.1 µm, an electrode length (*L_e_*) of 5 µm and a channel width (*W*) of 780 µm (Figure 1d). Contacts were then functionalized with a self‐assembled monolayer (SAM) of 2,3,4,5,6‐pentafluorothiophenol (PFBT), as part of the approach to improve hole injection [26, 38]. As a semiconductor, we used the small molecule/polymer blend C_8_‐BTBT:C_16_IDT‐BT, which has been repeatedly demonstrated to afford a field‐effect mobility in the range of 10 cm^2^ V^−1^ s^−1^ in OFET architectures characterized by long channel and overlap lengths [26, 39, 40, 41]. In the work of T. Losi et al., this material was also integrated into short‐channel transistors (*L_C_* = 1.3 µm), demonstrating the viability of using such a high‐performing blend in downscaled devices [26]. The semiconductor solution was formulated at 10 g l^−1^ in a 1:4 *wt*.% ratio between C_8_‐BTBT and C_16_IDT‐BT, with a doping concentration of 3.5 mol% of C_60_F_48_, and deposited via spin‐coating, leading to a 70 nm thick film [26, 42]. The semiconductor and dopant chemical structures are presented in Figure 1c. Molecular doping of the blend with C_60_F_48_, in combination with the SAM on the electrodes, was fundamental to further reduce contact resistance and therefore to enable high transconductance values while downscaling the transistor dimensions [26]. In particular, as reported in Ref. [26], a doping level equal to 3.5 mol% of C_60_F_48_ corresponds to the best trade‐off between an increase in off‐current and a reduction in contact resistance. The dielectric had to be developed balancing two conflicting needs. On the one hand, a high areal capacitance is needed to reduce the operational voltages, which can be achieved by thinning the dielectric layer. On the other hand, low gate leakage current and robustness during gate electrode processing must be maintained, which is particularly challenging in solution‐based processes. We used a bi‐layer structure formed by 30 nm of Teflon AF2400 deposited by spin‐coating and a thicker 120 nm film of Parylene‐C deposited by chemical vapour deposition, leading to a nominal areal capacitance *C_diel._* of 15.9 nF cm^−2^. Teflon AF2400 is known to form an ideal interface for charge transport with C_16_IDTBT:C_8_‐BTBT doped with C_60_F_48_ [38]. Parylene‐C, instead, has the advantage to form conformal films characterized by low leakage currents [8], and it is chemically robust and insoluble in many common processing solvents. Moreover, pinholes misalignment in the two dielectric layers ensures further reduction of defectivity. To complete the devices, top gate micron‐scale electrodes were patterned by fs‐laser sintering, directly over the transistor channel, keeping minimal overlaps with respect to the source and the drain bottom electrodes. The fs‐patterned gate contacts are characterized by a thickness (*t_g_*) of ∼ 70 nm and an electrode length (*L_g_*) of about ∼ 2 µm, leading to overlap lengths in the range of ∼ 0.4 µm (Figure 1d). Using a parallel plate model, we estimated total gate capacitances (*C_g tot_* = *C_g(ch)_* + *C_g(ov)_*) to be in the range of ∼ 0.18 pF – 0.25 pF, with the uncertainty related to the approximations adopted [11, 27].

**FIGURE 2:**
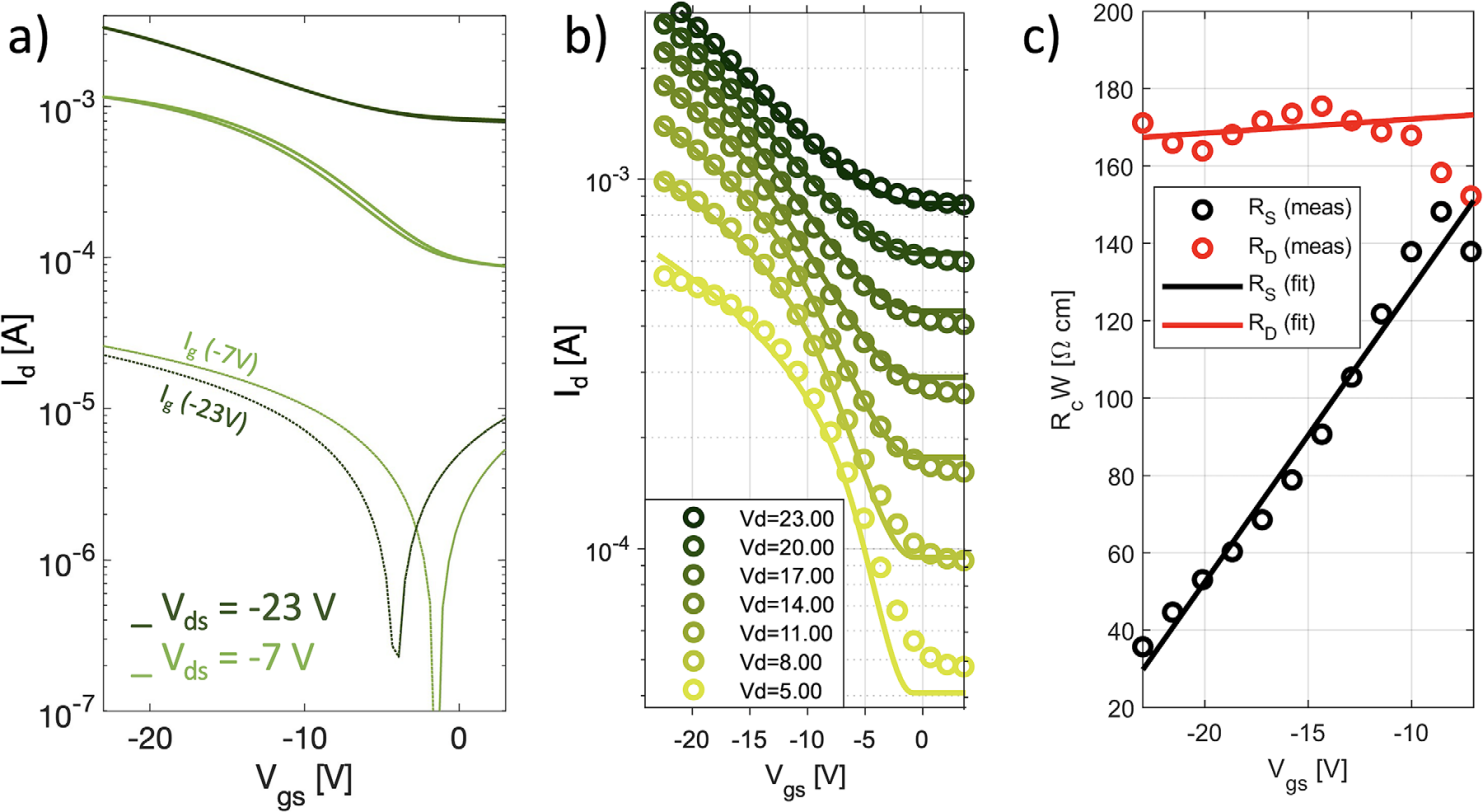
(a) Transfer curves measured at *V_ds_* = −7 V and at *V_ds_* = −23 V of a short‐channel device (*L_C_* = 1.1 µm) based on doped C_16_IDTBT:C_8_‐BTBT with a fs‐laser sintered gate electrode (*L_ov_* = 0.4 µm). (b) OFET measurements at different drain voltages (circles) and model fit (continuous lines). (c) Extraction of contact resistance as a function of *V_gs_*.

We measured the DC transfer characteristic curves of the transistors at *V_ds_* = −7 V and at *V_ds_* = −23 V, as presented in Figure 2a. An example of the output characteristic is reported in Figure S2. From Figure 2a it is possible to notice a strong unipolar p‐type behavior of the devices. Despite the relatively low dielectric thickness (∼ 150 nm), leakage currents are two orders of magnitude lower than transistor on‐currents, indicating robustness to the fs‐laser sintering of the gate. The high off‐current at zero drain voltage can be ascribed to the combination of the molecular doping level used and the short channel length of the devices. While such low on‐off current ratio is detrimental for logic applications, it is acceptable for RF analog designs where transistors are always biased in the on‐state. More attention should be made to RF applications where the transistors explore both states, as in rectifiers, since an increased off‐state current increases the rate at which the capacitor discharges, reducing the rectified output voltage. This reduction can be compensated by increasing the size of the rectifier capacitor.

In order to properly extract device DC parameters and to enable the simulation of the device AC behavior, we resorted to fitting with known physical OFET models. The DC characteristic curves were fitted according to the channel equation of a thin‐film transistor including the presence of contact resistance [43]:

(2) $$I_{d}=\frac{W}{L_{C}}\;\mu_{0}C_{diel.}\frac{{\left(V_{s}-I_{d}R_{s}-V_{T}-V_{g}\right)}^{\left(\gamma +2\right)}-{\left(V_{d}+I_{d}R_{d}-V_{T}-V_{g}\right)}^{\left(\gamma +2\right)}}{\gamma +2}\left(1+\lambda \left(V_{s}-V_{d}\right)\right)+I_{off},$$

where *I_d_* is the channel current, *μ_0_* is the static mobility of the device, *V_s_*, *V_g_* and *V_d_* are the potentials at the source, gate and drain respectively, *γ* is the mobility enhancement factor, *λ* is the channel length modulation factor and *I_off_* is the off‐current, which is a function of the source‐drain voltage. *R_s_* and *R_d_* are the source and drain contact resistances. Equation (2) is implicit; therefore, to extract all the parameters, first the equation without contact resistance (*R_s_* = *R_d_* = 0) is used to analytically obtain the model parameters, after which (2) is solved numerically to obtain the contact resistance. The first parameter to be extracted is *λ*, which models the channel length modulation and thus the output resistance, and can be found from the output OFET characteristic. By computing *r_0_* = (*∂V_ds_*/∂*I_d_*) in the saturation regime, *λ* can be found by converting from *r_0_* to *λ*, using the equation *λ* = 1/(*I_d_r_0_* – *V_d_*), which for our devices results in *λ* = 0.017 V^−1^. The following parameters to be extracted are *γ* and *V_T_*, which are obtained using the *H*_VG_ function [44], given by

(3) $$H_{VG}\left(V_{g}\right)=\frac{\int_{V_{T}}^{V_{g}}I_{d}\left(V_{g}\right)dV_{g}}{I_{d}\left(V_{g}\right)}\;$$

where the lower bound of the integration (*V_T_*) here is chosen as 1 V. Due to the high off‐current of the devices, *I_off_* is first subtracted from the measured drain current before computing the integral. Using this technique, it can be found that *γ*, which describes the increased mobility at higher gate overdrive voltages, is independent of the drain voltage (*γ* = 0.08), but the threshold voltage changes linearly with *V_d_* due to drain‐induced barrier lowering (DIBL) [45], which can be modelled as *V_T_* = 1.1 – 0.05*V_ds_* V. Filling in all extracted parameters in Equation (2) and again subtracting the off‐current leaves only *μ_0_* unknown. Therefore, *μ*_0_ can be found from the DC measurement points above the threshold *V_T_* as:

(4) $$\mu_{0}=\frac{\left(I_{d}\left(V_{g},V_{d}\right)-I_{off}\right)/W}{L_{C}}\;C_{diel.}\frac{{\left(V_{s}-I_{d}R_{s}-V_{T}-V_{g}\right)}^{\left(\gamma +2\right)}-{\left(V_{d}+I_{d}R_{d}-V_{T}-V_{g}\right)}^{\left(\gamma +2\right)}}{\gamma +2}\left(1+\lambda \left(V_{s}-V_{d}\right)\right)$$

where *I_d_* (*V_g_*,*V_d_*) is the measured DC current for a given gate and drain voltage. Due to the first order approximation that is still being considered, i.e. *R_s_* = *R_d_* = 0, the mobility is underestimated with this method at high channel currents, therefore the maximum mobility is chosen for the model, which for our devices gives *μ_0_* = 0.6 cm^2^ V^−1^ s^−1^.

Unlike conventional transistors with a low doping level, the off‐current of the OFETs shows a quadratic behavior with decreasing drain voltage, even if the device is biased in the off state (*V_gs_* < 0 V). This effect indicates that the drain voltage can induce a channel by itself, behaving like a diode. Therefore, in this work, the off‐current is modelled with two parameters: *η*, which is the “efficiency” of the diode compared to the channel generated by the gate, and the other is *R_off_*, modelling the resistance of the channel in off‐state. The equation for the off‐current therefore becomes:

(5) $$\begin{array}{cc} I_{off} & =\eta \frac{W}{L_{C}}\mu_{0}C_{diel.}\frac{{\left(V_{s}-V_{T}-V_{d}\right)}^{\left(\gamma +2\right)}}{\gamma +2}\left(1+\lambda \left(V_{s}-V_{d}\right)\right)\\ & \;+\frac{V_{s}-V_{d}}{R_{off}} \end{array}$$

By fitting Equation (5), we found *η* = 0.28 and *R_off_* = 209 kΩ. Finally, due to the implicit nature of the contact resistance, *R_s_* and *R_d_* are found using a least‐squares fit, assuming a linear dependence with the gate voltage. Figure 2b shows the model fitting for a single device at various *V_ds_*, demonstrating a good matching with the experimental data. Figure 2c shows instead the extraction of width‐normalized contact resistance, proving the linear dependence with *V_gs_*. A value as low as ∼ 200 Ω cm is derived in agreement with Ref. [26], highlighting the critical role of molecular doping with C_60_F_48_ in the downscaling of the device. From the transfer curves, we extracted a DC saturation channel transconductance at *V_gs_* = *V_ds_* = ‐23 V as *g_m_* = (*∂I_d_*/∂*V_gs_*)*_Vds_*. For all the fabricated OFETs the *g_m_* values found fall in the range of 150 µS – 200 µS, with deviations ascribable to device‐to‐device performance variation.

We then turned to the AC behavior of the transistors. Using Equation (1) and considering the previously extracted *g_m_* and a total gate capacitance of 0.21 pF (average of the nominal values considering a parallel plate model), a theoretical *f_T_* in between 110 MHz and 150 MHz is predicted, placing the OFETs well within VHF bandwidth.

**FIGURE 3:**
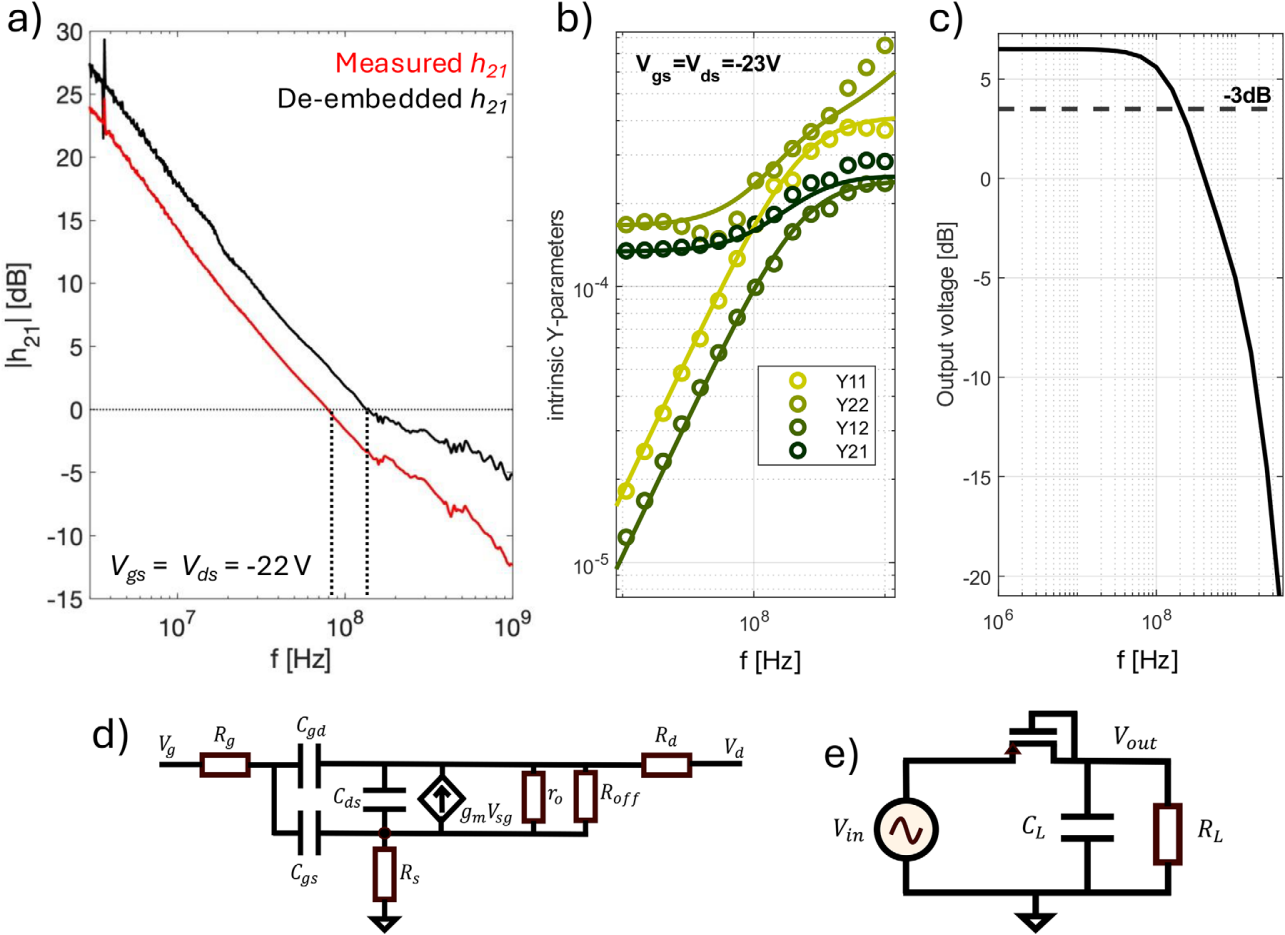
(a) Modulus of the *h_21_* parameter, before (red curve) and after (black curve) de‐embedding, of the best performing OFET obtained at *V_gs_* = *V_ds_* = ‐22 V. (b) Y‐parameters derived for an OFET having *f_T_* above 100 MHz (circles) and model fit (continuous lines). (c) Simulation of a half‐wave rectifier implementing the OFET relative to panel (b). (d) Circuital scheme of the AC model used for the Y‐parameter simulation of the fabricated transistors. (e) Circuital diagram for the half‐wave rectifier, where the load resistance and capacitance adopted for the simulation are *R_L_* = 100 kΩ and *C_L_* = 2 nF, respectively. The amplitude of the input sinewave *V_in_* was set to −23 V.

The experimental *f_T_* was determined by performing S‐parameters characterization to obtain the extrinsic hybrid parameter *h_21_* (Figure 3a), with the methodology reported by M. Giorgio et al. [46] (Figure S3a). These measurements were performed in air, without any encapsulation layer. The extrinsic *h_21_* was de‐embedded considering the parasitism of pads and interconnections as an additional parallel contribution to the transistors (Figure S3b,c). Further details about the model used for de‐embedding can be found in Ref. [27, 47]. From the intrinsic *h_21_*, the value of *f_T_* can be obtained by looking at the crossing point with the zero axis (*h_21_* (*f_T_*) = 0). A transition frequency as high as 135 MHz was measured at *V_gs_* = *V_ds_* = ‐22 V from the best‐performing OFET based on doped C_16_IDTBT:C_8_‐BTBT (Figure 3a). Figure S4 presents the *h_21_* parameter of three additional OFETs fabricated in this work having similar *f_T_*, while Table S1 reports a detailed comparison of extracted and theoretical parameters across multiple devices. To assess the physical validity of the AC characterization, the transistor Y‐parameters were also computed. From the value of the Y_21_ parameter at low frequency, a small‐signal channel transconductance as high as 170 µS is extracted for the best performing transistor, in agreement with the DC value obtained from the transfer characteristic (Figure S5a). From the imaginary part of the de‐embedded Y_11_ parameter, a value of total gate capacitance as low as ∼ 0.2 pF is derived (Figure S5b), in agreement with the nominal value computed using a simple parallel plate model.

The demonstration of VHF operation of the reported OFETs prompted us to assess their potential for use in electronic components operating in this frequency range through circuit simulation. To allow simulations, the device AC characteristics were modeled according to the method described by Torres‐Torres and Murphy‐Arteaga [48], except for the de‐embedding of the current path through the substrate, which is expected to be insignificant in the devices used due to the low conductivity of AlN (Figure 3d). After the de‐embedding procedure, the contacts resistance is determined for a device biased at *V_gs_* = ‐23 V and *V_ds_* = 0 V, to minimize the effect of the channel, resulting in *R_g_* = 2.13 kΩ, *R_s_* = 651 Ω and *R_d_* = 491 Ω. Next, from the measurements of a device biased in the on‐state (*V_gs_* = *V_ds_* = ‐23 V), the contact resistance is removed, allowing for extraction of the parasitic capacitances (*C_gs_* = 0.11 pF, *C_gd_* = 0.17 pF, *C_ds_* = 0.017 pF), an output resistance of 2.5 kΩ and the channel transconductance of 185 µS. The AC model of the device was implemented in Cadence Virtuoso to simulate the Y‐parameters. Figure 3b shows that the AC model and the experimental measurements of all Y‐parameters match closely. Furthermore, the *f_T_* values calculated from the *h_21_* graph are identical between the model and the measurements (Figure S6). Having also validated the AC model of the VHF OFET, by combining DC and AC models, we could simulate a simple half‐wave rectifier based on it (Figure 3e). Rectifiers are a fundamental component in wireless communication used, for example, in passive RFID tags to rectify an incoming RF wave and provide DC power to the tag chip. Results of the simulation indicate that the predicted −3 dB cut‐off frequency of the rectifier is 200 MHz (Figure 3c).

## 3 Conclusions and Future Perspectives

In contrast to the widespread view that organic microelectronics is bounded to low‐frequency operation, in this work we demonstrated direct‐written, solution‐fabricated VHF organic field‐effect transistors based on a small molecule/polymer semiconductor blend (C_16_IDT‐BT:C_8_‐BTBT). A transition frequency up to135 MHz was obtained at −22 V, which corresponds to a record value for p‐type OFETs and establishes a new benchmark of *f_T_*/*V^2^* for VHF organic transistors with *f_T_* > 100 MHz equal to 0.28 MHz V^−2^. This result was achieved applying a set of strategies aimed at overcoming technological barriers for very‐high frequency performance for OFETs such as: the use of high thermally conductive substrates and of a relatively thin organic dielectric to mitigate self‐heating degradation, the use of high doping levels to reduce contact resistance, and the minimization of gate parasitism by the reduction of geometrical overlaps with bottom electrodes (*L_ov_* of 0.4 µm). With the use of fs‐laser direct‐writing, we demonstrated mask‐less direct metal sintering of high‐resolution gate contacts directly on thin organic dielectrics. With this strategy, a gate width‐normalized capacitance as low as 2.5 pF cm^−1^ has been obtained, which enabled VHF operation in the realized transistors. The relevance of the experimental results is further highlighted by circuit‐level simulations, which show that the fabricated OFETs could potentially enable half‐wave rectifiers with a ‐3 dB cut‐off frequency of 200 MHz. This finding marks a significant advancement and establishes a solid foundation for future fabrication of solution‐fabricated UHF OFETs and circuits that would pave the way for the development of cheap wireless organic microelectronics.

## 4 Methods

### 4.1 Materials and Solution Preparation

C_16_IDT‐BT was synthesised as previously reported, with a M_w_ of 157 Kg mol^−1^ and Ð of 2.4 as measured by gel permeation chromatography (chlorobenzene, 80 °C) against polystyrene standards. C_8_‐BTBT (≥ 99% (HPLC), Sigma Aldrich) and C_16_IDT‐BT were both dissolved in chlorobenzene and tetralin (both from Sigma Aldrich) in 1:1 proportion at concentration of 10 g l^−1^ at 80°C. Then, the C_8_‐BTBT:C_16_IDT‐BT blend solution was prepared by mixing those of the two semiconductors in 1:4 ratio. C_60_F_48_ (provided by Olga Boltalina, Department of Chemistry of the Colorado State University) was dissolved at room temperature in Chlorobenzene at concentration of 3 g l^−1^ by stirring for 3 h. To dope the transistors, a given amount of C_60_F_48_ was added to the blend solution to reach a doping level of 3.5 mol% (calculated considering the molecular weight of the small molecule and that of the repeating unit of the polymer).

The SAM solution was prepared by mixing 7 µL of 2,3,4,5,6‐pentafluorothiophenol (PFBT) (97%, Sigma–Aldrich) with 10 mL of isopropyl alcohol (Sigma–Aldrich) for a final concentration of 0.0007 *v*/*v* at room temperature and stirred for 1 h.

For the dielectric, poly[4,5‐difluoro‐2,2‐bis(trifluoro‐methyl)‐1,3‐dioxole‐co‐tetrafluoro‐ethylene] (Teflon AF2400) was dissolved in Fluorinert FC‐40 (Sigma Aldrich) at concentration of 10 g l^−1^ at 80°C under stirring conditions. Poly(chloro‐p‐xylene)‐C (Parylene‐C dimer) was purchased from Specialty Coating Systems. The total areal capacitance of the bi‐layer dielectric was calculated using the literature values of dielectric constants for both materials equal to 2.1 for Teflon and 2.9 for Parylene‐C.

The silver nanoparticle ink (NPS‐L) was purchased from Harima Chemicals and filtered with 0.45 µm PTFE filters.

### 4.2 OFETs Fabrication

Bottom‐contacts top‐gate transistors were fabricated on aluminum nitride substrates (AlN) purchased from MARUWA CO., LTD. with an average surface roughness Ra ≤ 1 nm. A 300 nm thick layer of silicon dioxide (SiO_2_) was deposited by sputtering (Leybold‐Heraeus LH Z400 MS system). Source and drain electrodes were defined by maskless photolithography, evaporating 30 nm of gold with a 3 nm thick chromium adhesion layer. Gold contacts were then cleaned by sonication with acetone and isopropyl alcohol (Sigma–Aldrich) for 10 and 5 min, respectively, followed by an oxygen plasma treatment (100 W for 5 min). Then, a self‐assembled monolayer was formed on gold by immerging the samples for 15 min in the PFBT solution. The blend was deposited by spin‐coating in a two‐step procedure (500 rpm for 10 s and 1500 rpm for 30 s). Then the film is dried on a hotplate at 120°C for 1 min and 45 s. Teflon AF2400 was spin‐coated on top at 1000 rpm for 10 s and 3000 rpm for 60 s. Then, Parylene‐C was deposited by CVD with a SCS Labcoater 2‐PDS2010 system. For the gate fabrication, Ag NPS‐L ink was deposited by spin‐coating at 7000 rpm for 300 s, yielding a 70 nm thick layer. Laser sintering was then performed using few mW beam power, wavelength of 1030 nm, 50× optics, repetition rate of 74 MHz and 20 µm s^−1^ scanning speed. After that, the non‐sintered parts are washed away with o‐Xylene (Sigma–Aldrich) and dried with a nitrogen flux. The silver top gate electrode is electrically connected to the corresponding bottom gold interconnection pad with through‐dielectric vias realized by fs‐laser ablation performed with wavelength of 515 nm, 50× optics, repetition rate of 500 kHz and 0.1 mm s^−1^ scanning speed.

### 4.3 Static and Dynamic Electrical Characterization

Transfer and output characteristic curves were acquired with a Semiconductor Parameter Analyzer Agilent B1500A in glovebox (oxygen and water levels below 1 ppm). The S‐parameters characterizations were carried out in air using an Agilent Vector Network Analyzer N5231A with GS/SG |Z|‐probes purchased from Cascade Microtech and an Agilent B2912A Source Meter. Before any AC measurement, a calibration with a Short‐Open‐Load‐Through (SOLT) procedure was applied and corrected with a 12‐term error model. This step is needed to remove capacitive contributions and non‐idealities relative to the measurement setup itself and to move the calibration plane at the probe tips [46].

## Author Contributions

**Tommaso Losi**: conceptualization, investigation, writing – original draft, methodology, writing – review and editing. **Kyle van Oosterhout**: investigation, methodology, validation, formal analysis, writing – review and editing. **Martino Cambiaggio**: investigation, writing – review and editing, validation, methodology. **Martin Heeney**: investigation, supervision, resources, writing – review and editing. **Qiao He**: investigation, writing – review and editing, methodology. **Hans Kleemann**: methodology, investigation, writing – review and editing. **Alessandro Luzio**: investigation, writing – review and editing, methodology, supervision. **Eugenio Cantatore**: resources, supervision, writing – review and editing, methodology, funding acquisition. **Mario Caironi**: writing – original draft, conceptualization, funding acquisition, methodology, writing – review and editing, project administration, supervision, resources.

## Funding

This work was funded by the European Space Agency under the Discovery programme related to the OSIP Campaign “Open Discovery Ideas Channel” I‐2023‐07677 “GHioZa”. This work was partially supported by the European Union under the Horizon Europe grant 101161032 “GRETA” and by King Abdullah University of Science and Technology (KAUST) under Award No. ORFS‐CRG12‐2024‐6443.

## Conflicts of Interest

The authors declare no conflicts of interest.

## Supporting information




**Supporting File**: advs77513‐sup‐0001‐SuppMat.docx.

## Data Availability

The data that support the findings of this study are available in the supplementary material of this article.

## References

[1] L. Portilla , K. Loganathan , H. Faber , et al., “Wirelessly Powered Large‐Area Electronics for the Internet of Things,” Nature Electronics 6 (2023): 10–17, 10.1038/s41928-022-00898-5.

[2] W. Shi , Y. Guo , and Y. Liu , “When Flexible Organic Field‐Effect Transistors Meet Biomimetics: A Prospective View of the Internet of Things,” Advanced Materials 32, no. 15 (2020): 1901493, 10.1002/adma.201901493. 31250497

[3] M. Catacchio , M. Caputo , L. Sarcina , et al., “Spiers Memorial Lecture: Challenges and Prospects in Organic Photonics and Electronics,” Faraday Discussions 250 (2024): 9–42, 10.1039/d3fd00152k. 38380468

[4] G. Dufil , I. Bernacka‐Wojcik , A. Armada‐Moreira , and E. Stavrinidou , “Plant Bioelectronics and Biohybrids: The Growing Contribution of Organic Electronic and Carbon‐Based Materials,” Chemical Reviews 122, no. 4 (2022): 4847–4883, 10.1021/acs.chemrev.1c00525. 34928592 PMC8874897

[5] A. Kyndiah , G. Z. Zemignani , C. Ronchi , et al., “Non‐Invasive Action Potential Recordings Using Printed Electrolyte‐Gated Polymer Field‐Effect Transistors,” Nature Communications 16, no. 1 (2025): 8143, 10.1038/s41467-025-63484-1. PMC12398533 40885735

[6] W. Li , C. Zhang , D. Lan , W. Ji , and Y. Wang , “Solution Printing of Electronics and Sensors: Applicability and Application in Space,” Advanced Engineering Materials 24, no. 9 (2022): 2200173, 10.1002/adem.202200173.

[7] E. Stucchi , A. D. Scaccabarozzi , F. A. Viola , and M. Caironi , “Ultraflexible All‐Organic Complementary Transistors and Inverters Based on Printed Polymers,” Journal of Materials Chemistry C 8, no. 43 (2020): 15331–15338, 10.1039/d0tc03064c.

[8] E. Stucchi , G. Dell'Erba , P. Colpani , Y. H. Kim , and M. Caironi , “Low‐Voltage, Printed, All‐Polymer Integrated Circuits Employing a Low‐Leakage and High‐Yield Polymer Dielectric,” Advanced Electronic Materials 4, no. 12 (2018): 1800340, 10.1002/aelm.201800340.

[9] F. A. Viola , J. Barsotti , F. Melloni , et al., “A sub‐150‐Nanometre‐Thick and Ultraconformable Solution‐Processed All‐Organic Transistor,” Nature Communications 12, no. 1 (2021): 5842, 10.1038/s41467-021-26120-2. PMC8494881 34615870

[10] G. E. Bonacchini , C. Bossio , F. Greco , et al., “Tattoo‐Paper Transfer as a Versatile Platform for All‐Printed Organic Edible Electronics,” Advanced Materials 30, no. 14 (2018): 1706091, 10.1002/adma.201706091. 29460421

[11] A. Perinot , B. Passarella , M. Giorgio , and M. Caironi , “Walking the Route to GHz Solution‐Processed Organic Electronics: A HEROIC Exploration,” Advanced Functional Materials 30, no. 20 (2020): 1907641, 10.1002/adfm.201907641.

[12] B. Latré , B. Braem , I. Moerman , C. Blondia , and P. Demeester , “A Survey on Wireless Body Area Networks,” Wireless Networks 17, no. 1 (2011): 1–18, 10.1007/s11276-010-0252-4.

[13] H. H. Choi , K. Cho , C. D. Frisbie , H. Sirringhaus , and V. Podzorov , “Critical Assessment of Charge Mobility Extraction in FETs,” Nature Materials 17, no. 1 (2017): 2–7, 10.1038/nmat5035. 29255225

[14] O. Marinov and M. Jamal Deen , “Quasistatic Compact Modelling of Organic Thin‐Film Transistors,” Organic Electronics 14, no. 1 (2013): 295–311, 10.1016/j.orgel.2012.10.031.

[15] T. Zaki , S. Scheinert , I. Horselmann , et al., “Accurate Capacitance Modeling and Characterization of Organic Thin‐Film Transistors,” IEEE Transactions on Electron Devices 61, no. 1 (2014): 98–104, 10.1109/TED.2013.2292390.

[16] H. Klauk , “Will We See Gigahertz Organic Transistors?,” Advanced Electronic Materials 4, no. 10 (2018): 1700474, 10.1002/aelm.201700474.

[17] J. W. Borchert , R. T. Weitz , S. Ludwigs , and H. Klauk , “A Critical Outlook for the Pursuit of Lower Contact Resistance in Organic Transistors,” Advanced Materials 34, no. 2 (2022): 2104075, 10.1002/adma.202104075. 34623710 PMC11468869

[18] M. Waldrip , O. D. Jurchescu , D. J. Gundlach , and E. G. Bittle , “Contact Resistance in Organic Field‐Effect Transistors: Conquering the Barrier,” Advanced Functional Materials 30, no. 20 (2020): 1904576, 10.1002/adfm.201904576.

[19] U. Zschieschang , J. W. Borchert , M. Giorgio , et al., “Roadmap to Gigahertz Organic Transistors,” Advanced Functional Materials 30, no. 20 (2020): 1903812, 10.1002/adfm.201903812.

[20] T. Losi , Ł. Witczak , M. Łysień , et al., “High Frequency Solution‐Processed Organic Field‐Effect Transistors With High‐Resolution Printed Short Channels,” Advanced Functional Materials 33, no. 48 (2023): 2302656, 10.1002/adfm.202302656.

[21] G. Grau and V. Subramanian , “Dimensional Scaling Of High‐Speed Printed Organic Transistors Enabling High‐Frequency Operation,” Flexible and Printed Electronics 5, no. 1 (2020): 014013, 10.1088/2058-8585/ab739a.

[22] S. Chung , K. Cho , and T. Lee , “Recent Progress in Inkjet‐Printed Thin‐Film Transistors,” Advanced Science 6, no. 6 (2019): 1801445, 10.1002/advs.201801445. 30937255 PMC6425446

[23] G. O. Nikiforov , D. Venkateshvaran , S. Mooser , et al., “Current‐Induced Joule Heating and Electrical Field Effects in Low Temperature Measurements on TIPS Pentacene Thin Film Transistors,” Advanced Electronic Materials 2, no. 12 (2016): 1600163, 10.1002/aelm.201600163.

[24] A. Fischer , P. Pahner , B. Lüssem , et al., “Self‐Heating Effects in Organic Semiconductor Crossbar Structures With Small Active Area,” Organic Electronics 13, no. 11 (2012): 2461–2468, 10.1016/j.orgel.2012.06.046.

[25] M. P. Klinger , A. Fischer , H. Kleemann , and K. Leo , “Non‐Linear Self‐Heating in Organic Transistors Reaching High Power Densities,” Scientific Reports 8, no. 1 (2018): 9806, 10.1038/s41598-018-27689-3. 29955076 PMC6023904

[26] T. Losi , F. A. Viola , E. Sala , et al., “Downscaling of Organic Field‐Effect Transistors based on High‐Mobility Semiconducting Blends for High‐Frequency Operation,” Small Methods 8, no. 12 (2024): 2400546, 10.1002/smtd.202400546. 39104287 PMC11671851

[27] A. Perinot , M. Giorgio , V. Mattoli , D. Natali , and M. Caironi , “Organic Electronics Picks Up the Pace: Mask‐Less, Solution Processed Organic Transistors Operating at 160 MHz,” Advanced Science 8, no. 4 (2021): 2001098, 10.1002/advs.202001098. 33643784 PMC7887599

[28] A. Mediano and F. J. Ortega‐Gonzalez , “Class‐E Amplifiers and Applications at MF, HF, and VHF: Examples and Applications,” IEEE Microwave Magazine 19, no. 5 (2018): 42–53, 10.1109/MMM.2018.2823238.

[29] F. H. Raab , R. Caverly , R. Campbell , et al., “HF, VHF, and UHF Systems and Technology,” IEEE Transactions on Microwave Theory and Techniques 50, no. 3 (2002): 888–899, 10.1109/22.989972.

[30] R. Caverly , G. Breed , W. H. Cantrell , et al., “Advancements at the Lower End: Advances in HF, VHF, and UHF Systems and Technology,” IEEE Microwave Magazine 16, no. 1 (2015): 28–49, 10.1109/MMM.2014.2367855.

[31] A. D. Scaccabarozzi , J. I. Basham , L. Yu , et al., “High‐Density Polyethylene—An Inert Additive With Stabilizing Effects on Organic Field‐Effect Transistors,” Journal of Materials Chemistry C 8, no. 43 (2020): 15406–15415, 10.1039/d0tc03173a.

[32] D. Nava , Y. Shin , M. Massetti , et al., “Drastic Improvement of Air Stability in an n‐Type Doped Naphthalene‐Diimide Polymer by Thionation,” ACS Applied Energy Materials 1, no. 9 (2018): 4626–4634, 10.1021/acsaem.8b00777. 30288490 PMC6166998

[33] A. Perinot and M. Caironi , “Accessing MHz Operation at 2 V With Field‐Effect Transistors Based on Printed Polymers on Plastic,” Advanced Science 6, no. 4 (2019): 1801566, 10.1002/advs.201801566. 30828529 PMC6382309

[34] M. Dutta , N. R. Das , B. Iñiguez , A. Kloes , and G. Darbandy , “Review of DC and AC Core Compact Models and Device Performance in Organic Transistors,” Journal of Applied Physics 139, no. 1 (2026): 010701, 10.1063/5.0303946.

[35] J. W. Borchert , U. Zschieschang , F. Letzkus , et al., “Flexible Low‐Voltage High‐Frequency Organic Thin‐Film Transistors,” Science Advances 6, no. 21 (2020): aaz5156, 10.1126/sciadv.aaz5156. PMC7314562 32671209

[36] T. Izawa , E. Miyazaki , and K. Takimiya , “Molecular Ordering of High‐Performance Soluble Molecular Semiconductors and Re‐evaluation of Their Field‐Effect Transistor Characteristics,” Advanced Materials 20, no. 18 (2008): 3388–3392, 10.1002/adma.200800799.

[37] W. Zhang , J. Smith , S. E. Watkins , et al., “Indacenodithiophene Semiconducting Polymers for High‐Performance, Air‐Stable Transistors,” Journal of the American Chemical Society 132, no. 33 (2010): 11437–11439, 10.1021/ja1049324. 20677750

[38] A. F. Paterson , A. D. Mottram , H. Faber , et al., “Impact of the Gate Dielectric on Contact Resistance in High‐Mobility Organic Transistors,” Advanced Electronic Materials 5, no. 5 (2019): 1800723, 10.1002/aelm.201800723.

[39] A. F. Paterson , Y. H. Lin , A. D. Mottram , et al., “The Impact of Molecular p‐Doping on Charge Transport in High‐Mobility Small‐Molecule/Polymer Blend Organic Transistors,” Advanced Electronic Materials 4, no. 10 (2018): 1700464, 10.1002/aelm.201700464.

[40] A. Basu , M. R. Niazi , A. D. Scaccabarozzi , et al., “Impact of p‐Type Doping on Charge Transport in Blade‐Coated Small‐Molecule:Polymer Blend Transistors,” Journal of Materials Chemistry C 8, no. 43 (2020): 15368–15376, 10.1039/d0tc03094e.

[41] A. F. Paterson , N. D. Treat , W. Zhang , et al., “Small Molecule/Polymer Blend Organic Transistors With Hole Mobility Exceeding 13 cm 2 V^−1^ s^−1^ ,” Advanced Materials 28, no. 35 (2016): 7791–7798, 10.1002/adma.201601075. 27374749

[42] A. F. Paterson , Next‐Generation Organic Blend Semiconductors for High Performance Solution‐Processable Field Effect Transistors (PhD thesis, Imperial College, 2017).

[43] O. Marinov , M. J. Deen , U. Zschieschang , and H. Klauk , “Organic Thin‐Film Transistors: Part I—Compact DC Modeling,” IEEE Transactions on Electron Devices 56, no. 12 (2009): 2952–2961, 10.1109/TED.2009.2033308.

[44] M. J. Deen , O. Marinov , U. Zschieschang , and H. Klauk , “Organic Thin‐Film Transistors: Part II—Parameter Extraction,” IEEE Transactions on Electron Devices 56, no. 12 (2009): 2962–2968, 10.1109/TED.2009.2033309.

[45] T. J. Yang , J. H. Kim , C. I. I. Ryoo , et al., “Analysis of Drain‐Induced Barrier Lowering in InGaZnO Thin‐Film Transistors,” IEEE Transactions on Electron Devices 70, no. 1 (2023): 121–126, 10.1109/TED.2022.3223642.

[46] M. Giorgio and M. Caironi , “Radio‐Frequency Polymer Field‐Effect Transistors Characterized by S‐Parameters,” IEEE Electron Device Letters 40, no. 6 (2019): 953–956, 10.1109/LED.2019.2909844.

[47] M. C. A. M. Koolen , J. A. M. Geelen , and M. P. J. G. Versleijen , “An Improved Deembedding Technique For On‐Wafer High‐Frequency Characterization,” Bipolar Circuits and Technology Meeting (IEEE, 1991).

[48] R. Torres‐Torres and R. Murphy‐Arteaga , “Straightforward Determination of Small‐Signal Model Parameters for Bulk RF‐MOSFETs,” in Proceedings of the Fifth IEEE International Caracas Conference on Devices, Circuits and Systems (ICCDCS) (IEEE, 2004), 14–18, 10.1109/ICCDCS.2004.1393345.

