# Supplementary material for: A Very‐High Frequency Solution‐Processed Organic Field‐Effect Transistor With Improved Voltage‐Normalized Frequency of Transition
Source: Adv Sci (Weinh). 2026 Sep 13:e77513. Online ahead of print. doi: 10.1002/advs.77513 (PMC13572828; doi:10.1002/advs.77513)
Supplement: Supplementary file 1 [file ADVS-9999-e77513-s001.docx]

Supporting Information

**A Very-High Frequency Solution-Processed Organic Field-Effect Transistor with Improved Voltage-Normalized Frequency of Transition**

*Tommaso Losi, Kyle Van Oosterhout, Martino Cambiaggio, Martin Heeney, Qiao He, Hans Kleemann, Alessandro Luzio, Eugenio Cantatore, Mario Caironi**

**Figure S1**

a) Device architecture used for bias stability characterizations. The gate in PEDOT:PSS was ink-jet printed on top of the channel in full-overlap. The channel length and channel width of the devices are *L_C_* = 2 µm and *W* = 2 mm. b) Cyclic measurements performed on a single transistor and c) bias stress performed at the maximum operating voltages. All the measurements were performed in air.

**Figure S2**

Example of out-put characteristic curve of a doped C_8_-BTBT:C_16_IDT-BT transistor with *L_C_* 1.5 μm of and with *L_ov_* of 0.35 μm. It can be observed that drain current does not saturate properly with increasing drain voltage, likely due to short-channel effects. ^[1]^

**Figure S3**

a) Example of S-parameter characterization in saturation regime (*V_gs_* = *V_ds_* = -23 V) of a downscaled OFET based on doped C_8_-BTBT:C_16_IDT-BT with low overlaps (*L_C_* of 1.1 μm and *L_ov_* of 0.4 μm). b) S-parameter measurements of an open structure under same bias conditions (*V_gs_* = *V_ds_* = -23 V) for OFETs Y-parameters de-embedding. c) Schematic cross-section of OFET and open structure. d) Scheme of OFET’s source/drain and open layout used during the photolithography step. To perform the de-embedding procedure, the Y-parameters of the transistors and the ones of the open are first computed. Then, the Y-parameters of the open are subtracted from the ones of the transistors, obtaining the de-embedded OFET Y-parameters. After that, it is possible to compute the de-embedded S-parameters and therefore the de-embedded hybrid *h_21_*.

**Figure S4**

Intrinsic *h_21_* parameters for three different short-channel transistors with laser sintered gates based on doped C_8_-BTBT:C_16_IDT-BT blend. The corresponding *f_T_* are: a) 115 MHz (at *V_gs_* = *V_ds_* = -23 V), b) 98 MHz (at *V_gs_* = *V_ds_* = -21 V) and c) 105 MHz (at *V_gs_* = *V_ds_* = -23 V).

**Figure S5**

The Y_21_ parameter is related to the channel transconductance of the transistor as: Y_21_ = *g_m_* - *i*2π*fC_gd_*. Therefore, the value of *g_m_* can be extracted either considering the real part Y_21_ or its value at low frequencies. In the panel a), an example of derivation of transconductance from the Y_21_ parameter is shown. The Y_11_ parameter is instead related to the total gate capacitance of the transistor as: Y_11_ = *i*2π*f* (*C_gs_* + *C_gd_*). Hence, *C_g tot_* can be directly obtained doing a linear fitting of Y_11_ with respect to the frequency. In the panel b), an example of extraction of gate capacitance is shown, fitting the Y_11_ parameter in a frequency range from 1 MHz up to 70 MHz.

**Figure S6**

Comparison between experimental (continuous black curve) and simulated (dotted black curve) hybrid parameter *h_21_* for one of the OFET fabricated in this work.

**Table S1**

Summary table of 4 VHF devices fabricated in this work including: DC extracted *g_m_*, Y-parameter derived *g_m_*, estimated and measured *C_g tot_*, and estimated and measured *f_T_*.

DC extracted *g_m_* was computed as *g_m_* = (∂*I_d_*/∂*V_gs_*)*_Vds_*, where *V_gs_* and *V_gs_* are the static voltages applied and *I_d_* is the DC source-drain current. In AC, *g_m_* is extracted considering the real part of the Y_21_ parameter.

*C_g tot_* was theoretically estimated using a parallel-plate model as: *C_g_* = (*C_gd (ov)_* + *C_gs (ov)_*) + *C_ch_* = *C_diel_* (*A_ov_* + α) *A_ch_* where *C_gd (ov)_* and *C_gs (ov)_* are the capacitive contributions related to the overlap of the source and drain electrodes with the gate, *C_ch_* the capacitive contribution of the channel, *C_diel_* the dielectric capacitance per unit area, *A_ov_* the total overlap area of both source and drain electrodes, *A_ch_* the total channel area and α a parameter ranging from 2/3 to 1 according to the mobility dependence on the bias voltage. We also accounted for fringing fields from the top gate with an additional contribution equal to *C_fin_* = 2*t_ins_WC_diel_* where *t_ins_* is the thickness of the dielectric. In the table we included a range of theoretical *C_g tot_* from the minimum (with no fringing fields and α = 2/3) and maximum value (with fringing fields and α = 1). The AC estimation of capacitances from the Y-parameters is obtained by considering the Y_11_ parameter since Y_11_ = *i*2π*f* (*C_gd_* + *C_gs_*). Hence, *C_g tot_* can be directly obtained doing a linear fitting with respect to the frequency. The values below are obtained with a fitting of the OFET de-embedded Y_11_ parameter in a frequency range from 10 MHz up to 100 MHz. A good agreement between theoretical and experimental capacity values can be appreciated.

From the table it is possible to note that the measured *f_T_* falls well in the range of theoretical *f_T_* values computed using the DC *g_m_* and the theoretical *C_g tot_*.

| **Device n.** | ***f_T_***  **[MHz]** | ***Theoretical f_T_***  **[MHz]** | ***DC extracted* *g_m_***  **[µS]** | ***Y-parameter derived g_m_***  **[µS]** | | ***Theoretical C_g tot_***  **[pF]** | ***Y-parameter derived C_g tot_***  **[pF]** |
| --- | --- | --- | --- | --- | --- | --- | --- |
| 1 | 135 MHz | 151 - 105 | 180 µS | | 170 µS | 0.189 - 0.272 | 0.2 |
| 2 | 115 MHz | 143 - 98 | 160 µS | | 140 µS | 0.177 - 0.259 | 0.183 |
| 3 | 98 MHz | 109 - 76 | 135 µS | | 125 µS | 0.198 - 0.284 | 0.23 |
| 4 | 105 MHz | 155 - 73 | 195 µS | | 160 µS | 0.2 - 0.284 | 0.24 |

**References**

[1] Y. Taur and T. H. Ning, *Fundamentals of Modern VLSI Devices* (Cambridge University Press 2021), https://doi.org/10.1017/CBO9781139195065.
